# 

**Published:** 1888-08

**Authors:** 


CONTENTS:

ARTICLE I.—Professional Patents. By W. Storer How, D. D. S...... 145

" II.—The Management of Pulpless Teeth from the Standpoint of Daily Practice. By A. W. Harlan, M.D.,D.D.S... 157

" III.—Bichloride of Mercury in Dental Practice. By Louis Ottofy, D. D. S ........ 164

" IV.—Some Difficult Cases and their Treatment. By L. P. Haskell........ 168

" V.—Copper Amalgam ........ 173

" VI.—Peroxide of Hydrogen ........ 176

" VII.—South Carolina Dental Association........ 180

" VIII.—Micro-Organisms and the Teeth. By Dr. M. Bissell ..... 184

EDITORIAL.

The Tariff on Instruments, Books, and Drugs........ 187

The National Association of Dental Examiners........ 190

American Dental Association ........ 190

MONTHLY SUMMARY.

Amalgam in Contact with Gold ........ 191

Prejudice Against Amalgam ........ 192

